# Honouring COVID-19 restrictions: A qualitative study of the virtual asd diagnostic pathway in a uk nhs camhs service

**DOI:** 10.1192/j.eurpsy.2021.622

**Published:** 2021-08-13

**Authors:** C. Ecob, S. Olety, R. Lancaster, A. Harris

**Affiliations:** Heathy Young Minds, Pennine Care NHS Foundation Trust, Stalybridge, United Kingdom

**Keywords:** autism spectrum disorder, COVID-19, diagnosis, virtual

## Abstract

**Introduction:**

The Multi-Agency Autism Team (MAAT) diagnose Autism Spectrum Disorder (ASD) in children and young people using a multi-stage assessment process. In March 2020, the UK went into lockdown due to the COVID-19 pandemic, affecting the MAAT’s ability to continue their typical diagnostic pathway.

**Objectives:**

This qualitative study aimed to assess the effectiveness and feasibility of a virtual ASD diagnostic pathway.

**Methods:**

From March – September; one hundred detailed developmental history assessments were conducted over the telephone, fifteen socially-distanced BOSA (Brief Observation of Symptoms of Autism) assessments were piloted, twenty-five multi-disciplinary formulation meeting were held over a video platform, and sixty diagnosis feedback consultations were conducted via telephone or video call. Structured interviews were conducted with clinicians and service-users.

**Results:**

revealed that telephone developmental history assessments were generally preferable over face-to-face appointments, and video-based formulation meetings were effective, productive and resulted in higher clinician attendance. The qualitative data on feedback appointments was mixed. Clinicians felt that telephone appointments were less personable and ethical; whereas, video-based feedback appointments allowed for more empathy. However, the majority of service-users opted for tele-calls over video-calls for these appointments. Socially-distanced BOSAs obtained positive clinician feedback in general. Service-user feedback was mixed; some found the experience uncomfortable and unfamiliar, whilst others enjoyed the experience. Overall, service-users were content with the knowledge that it may support a diagnostic outcome for their child.

**Conclusions:**

We concluded that the overall experience of the virtual ASD diagnostic pathway was a positive and informative process, identifying opportunities for permanent change to the service.

